# Medicaid Expansion’s Spillover to the Criminal Justice System: Evidence from Six Urban Counties

**DOI:** 10.7758/rsf.2020.6.2.11

**Published:** 2020-07

**Authors:** CARRIE E. FRY, THOMAS G. MCGUIRE, RICHARD G. FRANK

**Affiliations:** Harvard University.; Department of Health Care Policy at Harvard Medical School.; Department of Health Care Policy at Harvard Medical School.

**Keywords:** Medicaid, expansion, recidivism, mental health, substance use disorder

## Abstract

Spillovers from the Affordable Care Act Medicaid expansion to other social-sector outcomes have received little attention. One that may be especially salient for public policy is the impact of expanded eligibility on jail-related outcomes. This study compares recidivism outcomes in three non-expansion counties to nearby expansion counties before and after Medicaid expansion. Using forty-eight months of arrest data from six urban county jails, we conduct comparative interrupted time series analyses to describe changes in the probability of rearrest and the number of arrests before and after Medicaid expansion. Consistent with previous literature, we find mixed results. In two case studies, Medicaid expansion is associated with decreased rates of recidivism. In the other, we find differential increases in jail-based recidivism after Medicaid expansion. We use contextual information from site visits and stakeholder interviews to understand the factors that may mediate and moderate the relationship between Medicaid expansion and return to jail.

Prior to the authorization of state options for Medicaid expansion, low-income adults without dependent children were ineligible for Medicaid in most states. This population over-lapped with the jail-involved population significantly. Both groups were predominantly young, low-income, minority, and male. In 2014, men made up 85.3 percent (Zeng 2018) and young adults (eighteen to thirty-four years old) made up 60 percent of jail-involved individuals (Regenstein and Rosenbaum 2014). Additionally, men of color are more likely to be jailed than their white counterparts—one in 106 white men were incarcerated in 2006 versus one in thirty-six Latino and one in seven African American men (Regenstein and Rosenbaum 2014). Given this overlap, early estimates suggested that 25 to 30 percent of those released from jail in a given year would enroll in Medicaid in expansion states (Regenstein and Rosenbaum 2014).

Although federal law permits Medicaid coverage to continue during an individual’s incarceration, forty-six states do not continue coverage (Morrissey et al. 2006). The vast majority of people released from jail thus have historically found themselves in the community without immediate access to health insurance of any kind (Wang et al. 2008). This is particularly problematic for those with mental illnesses given that Medicaid is the largest payer for treatment services for these diagnoses in the United States and an increasingly large payer for treatment for substance use disorders. Thus, disruption in coverage reduces the likelihood of receiving timely community-based behavioral health-care services after release from incarceration. Together, these conditions reinforce a cycle whereby jail-involved individuals return to the community with little support.

Jail-involved individuals also have higher rates of chronic physical health conditions (such as asthma and diabetes), communicable diseases (such as HIV and Hepatitis C), mental illnesses, and substance use disorders than the general population (Marks and Turner 2014). Estimates from 2007 through 2009 report that 63 percent of sentenced jail inmates meet diagnosable criteria for drug dependence or abuse (Bronson et al. 2017), and 64 percent have a diagnosable mental illness at booking or in the twelve months prior to arrest. (Regenstein and Rosenbaum 2014). Indeed, jail-involved individuals have a 14 percentage point higher rate of serious mental illness (SMI) than the general population (Steadman et al. 2009).

These high risks stem in large part from the conditions under which justice-involved people live and from their limited ability to obtain appropriate care for their health needs. For instance, one-third of inmates taking prescription drugs do not have access to necessary medication while in jail, and more than half (60 percent) of those who require routine blood testing had no testing while in jail (Regenstein and Rosenbaum 2014). Additionally, most jail-involved people do not have access to health-care services in the community due to gaps in the health-care safety net, a lack of health insurance coverage, or both.

Providing timely access to health-care services, particularly treatment for mental illnesses and substance use disorders, may be one way to reduce rates of reoffense. Indeed, recent evidence indicates that improved access to evidence-based treatment for mental and addictive illnesses can improve reentry outcomes (Patel et al. 2014). Other studies, however, find mixed results. Additionally, many of these studies were retrospective and used nonexperimental designs that do not account for selection into treatment, precluding reliable causal inference.

Early work found that recently released people with an SMI in King County, Washington, and Pinellas County, Florida, who obtained Medicaid coverage on release from prison were 16 percent less likely to be rearrested in the following year and spent more time in the community before rearrest (102 versus ninety-three days) than similar people who did not get Medicaid coverage (Morrissey et al. 2007). In contrast, a study using administrative records from 2006 to 2007 found that although expedited Medicaid enrollment for people with SMI released from Washington State’s prisons led to greater Medicaid enrollment and mental health service use, the intervention was not associated with reduced rates of recidivism at twelve (Morrissey, Domino, and Cuddeback 2016) or thirty-six months after release (Grabert et al. 2017).

In a more recent study using these same data in Washington, Marisa Domino and colleagues (2019) examine the relationship between access to timely mental health services (defined as those received within twelve months of being released) and rates of recidivism for individuals recently released from prison. The authors find that the receipt of such services was associated with an increased rate of recidivism, specifically for technical violations, at twelve months since release. Interpreting these results as causal is difficult, given that those more likely to access services may also be less likely to violate parole or reoffend.

The U.S. Government Accountability Office (GAO) studied reentry programs in Florida and Michigan. In Florida, it found that access to community services, including through Medicaid, on release from prison was not associated with any change in the likelihood in rearrest, overall. Some groups (black and older individuals) did experience a slight decrease in the likelihood of rearrest (Costopoulos et al. 2017). During the study period, however, Florida’s Medicaid program did not pay for substance use disorder treatment, which may have mitigated any potential effect (Costopoulos et al. 2017). Michigan’s implemented program consisted of prison “in-reach” sessions, health screenings, and connections to health-care services for those soon to be released from prison (Sartorius and Woodbury 2014). After the program was implemented, the recidivism rate fell by 18.2 percentage points for two-year parolees and 8.4 percentage points for one-year parolees who received these services, relative to their rates before the services were implemented. However, this study lacked a control group or a well-defined intervention (Sartorius and Woodbury 2014).

## THE ACA AND RECIDIVISM

The ACA’s coverage provisions (including Medicaid expansion) not only increased eligibility for previously ineligible populations, but also changed the type of behavioral health-care services that low-income adults have access to. Prior to the ACA, the public behavioral health system was funded predominantly by categorical Medicaid programs (typically excluding single childless adults from eligibility) and state and federal budgeted funding mechanisms (such as federal block grants). These funding mechanisms often did not require evidence-based treatment in specialty substance use disorder treatment programs, nor did they encourage adequate treatment capacity.

Since ACA enactment, treatment providers in expansion states are generally required to meet conditions of participation in Medicaid, which require greater capacity and provision of evidence-based behavioral health care. Additionally, federal legislation and regulations attempt to standardize Medicaid benefits for behavioral health-care services. Together, the Mental Health Parity and Addictions Equity Act of 2008 and the ACA require all Medicaid managed care plans to cover treatment services for mental illness and substance use disorder as essential health benefits, and at parity with medical and surgical benefits. The ACA also requires that all enrollees from the expansion population have coverage for mental health and substance use disorder care that is at parity with medical and surgical coverage.

Despite federal legislation and regulation to standardize these benefits, the fee-for-service behavioral health services provided by Medicaid vary by state, given that fee-for-service Medicaid programs are not subject to the provisions of the Mental Health Parity and Addiction Equity Act or the ACA (with the exception of expansion adults). For instance, forty-three of the fifty-one jurisdictions surveyed cover inpatient psychiatric hospitals stays, four states require a copayment for these services, and seventeen states limit these services (Kaiser Family Foundation 2018). Coverage of residential psychiatric services is even more variable—only twenty-three state Medicaid programs provide this benefit. Although all fifty states provide coverage for buprenorphine for the treatment of opioid use disorder (OUD), twenty-one require a copayment for this service and nineteen require a prior authorization (Kaiser Family Foundation 2018). Additionally, ten states (including Louisiana, in our study sample) do not cover methadone for OUD.

In addition to changes in Medicaid benefit design and covered services, the target populations of the ACA’s Medicaid expansion differ from those included in previous studies examining the relationship between access to health-care services and recidivism. Taken together, these imply that previous findings about Medicaid and recidivism may not generalize to the expansion population. In this study, we extend previous analyses to the Medicaid expansion population and provide one of the first quasi-experimental analyses of the relationship between gaining health insurance coverage and criminal justice outcomes. To do so, we compare recidivist outcomes among jail-involved individuals in three non-expansion counties with nearby expansion counties before and after Medicaid expansion.

## DATA AND EMPIRICAL METHODS

We examine the relationship between expanded Medicaid eligibility and recidivism with an intent-to-treat analysis that takes advantage of the plausibly exogenous variation provided by Supreme Court ruling in *NFIB v. Sebelius*, which gave states the option to expand their Medicaid program.^1^ We used forty-eight continuous months of individual-level booking and release dates from six urban county jails (three in expansion states and three in nearby non-expansion states) and comparative interrupted time series regression analysis to describe the level and trends of the rate of rearrest and the number of arrests before and after Medicaid expansion in expansion and non-expansion counties. We also estimate the impact of expanded Medicaid eligibility on rates of recidivism for the whole sample and for the largest racial-ethnic groups. Finally, in a qualitative analysis, we identify county-level reentry or diversion programs/policies that may explain the differential relationship by county pair (if any) between expanded Medicaid coverage and recidivism.

### County Selection and Characteristics

**Figure 1.**
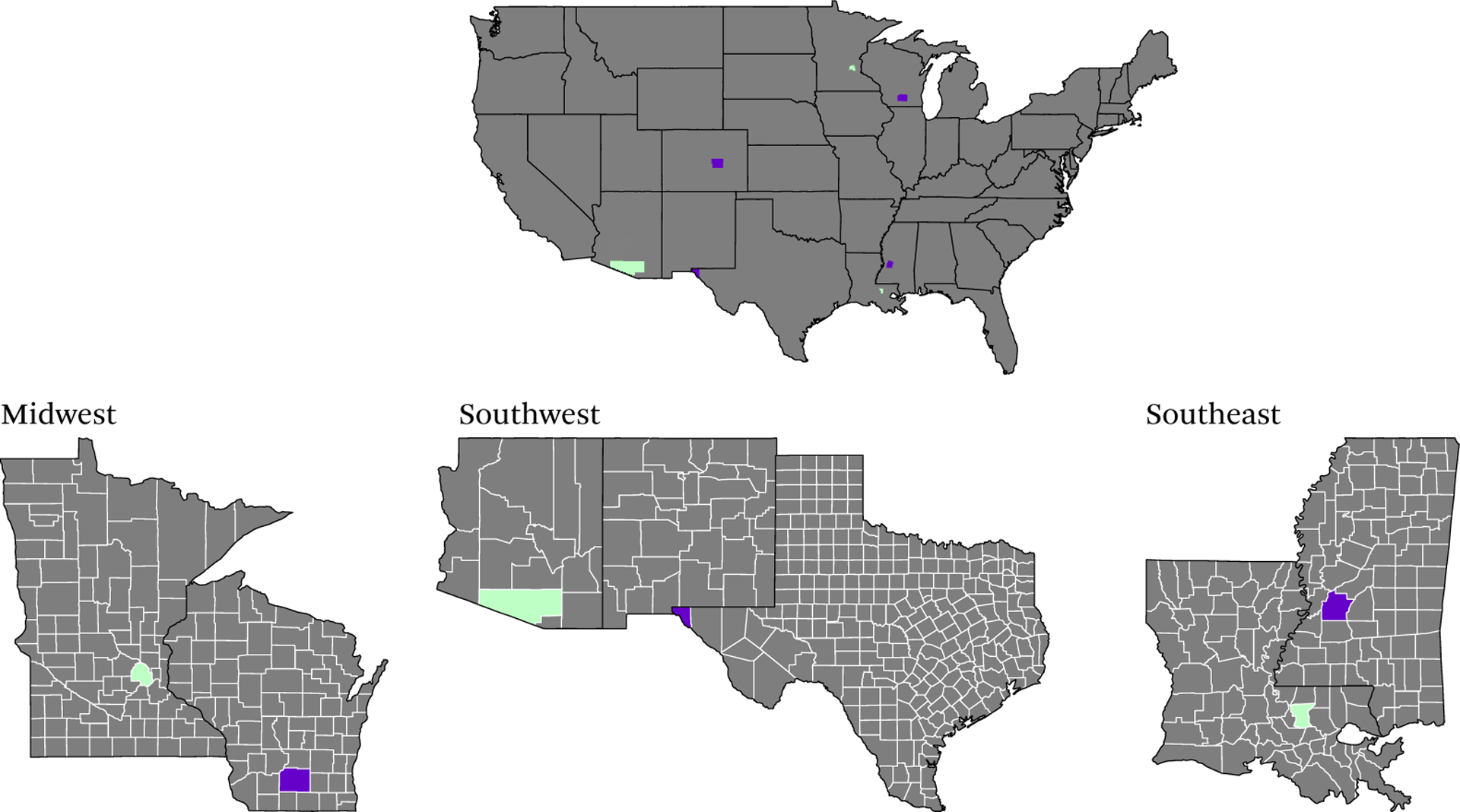
Study Counties *Source:* Authors’ analysis of county-level booking data. *Note:* Medicaid expansion counties are represented in the lighter shade; non-expansion counties are in the darker shade. Counties were chosen to provide geographic diversity across the United States and based on demographic similarity. Counties also had to have adequate volume in the county jail to provide adequate power for the analyses and had to be able to track recidivist outcomes for the entire study period. Counties in the Midwest are Hennepin County, Minnesota (expansion), and Dane County, Wisconsin (non-expansion). Counties in the Southwest are Pima County, Arizona (expansion), and El Paso County, Texas (non-expansion). Counties in the Southeast are East Baton Rouge Parish, Louisiana (expansion), and Hinds County, Mississippi (non-expansion).

We initially selected four county pairs—Hennepin County, Minnesota (expansion) and Dane County, Wisconsin (Midwest region); Pima County, Arizona (expansion) and El Paso County, Texas (Southwest region); East Baton Rouge Parish, Louisiana, (expansion) and Hinds County, Mississippi (Southeast region); and St. Louis, Missouri, and East St. Louis, Illinois. Three of these four pairs agreed to participate and provide data for our study; the St. Louis locales declined to participate (see figure 1). The initial choice of county pairs was based on a number of factors. First, geographic diversity was important. Because regions of the United States have distinct cultures, practices, and attitudes toward mental illness, substance abuse, and the criminal justice system, we included regions that represented these distinctions. We also wanted paired counties to be in adjacent or near-adjacent states. County pairs were also selected because they generally had comparable poverty rates, household income, rates of jailing, and approaches to pre-release coordination and eligibility determination (see table 1). It was also necessary to study county jails with enough volume to provide statistical power to detect the suspected effect size and data systems capable of tracking recidivism during our study period.

### Midwest

In the Midwest, both study counties had similar proportions of the population younger than eighteen—20 percent for Dane County and 22 percent for Hennepin County (see table 1). White, non-Hispanic people accounted for 85 percent of all residents in Dane and 77 percent in Hennepin (U.S. Census Bureau 2015). More than 95 percent of the Dane County population had graduated from high school at age twenty-five relative to 93 percent in Hennepin County (U.S. Census Bureau 2011). The poverty rates in the two counties were identical at 11 percent, the median household incomes in 2015 were $62,865 in Dane County and $65,834 in Hennepin (U.S. Census Bureau 2015). Both counties had population-adjusted jail rates in 2013 that were below the national average—122 for Dane and eighty-two for Hennepin (Vera Institute of Justice 2018).

In addition to similar demographic characteristics, both Wisconsin and Minnesota provide similar levels and types of Medicaid coverage for behavioral health conditions (see table A1^2^). Both counties have implemented programs to link justice-involved individuals with behavioral health-care services. In Dane County, Wisconsin, the jail has an Americorp volunteer on site three days a week to provide the jail’s inmates with enrollment assistance into BadgerCare (Wisconsin’s Medicaid program) prior to their release. Additionally, Dane County has a number of jail diversion initiatives, including electronic monitoring and reduced sentences for community program participation, that allows a person to remain in the community and receive community-based behavioral health services after adjudication.

Similarly, Hennepin County, Minnesota, has a countywide Integrated Access Team that identifies, screens, and refers the justice-involved for treatment and assures continuity of care whether the person is in or out of jail. Additionally, reentry staff work with the community-based case managers to connect inmates to medication assistance in the community. In addition to reentry programs, Hennepin County has diversion programs for people with behavioral health needs. In 2018 (which is after the study period in the Midwest county pair), Hennepin County opened a comprehensive social services facility that provides detoxification and mental health crisis services, employment counseling, and Medicaid eligibility assistance. All providers housed at the drop-in center accept Medicaid reimbursement for their services.

However, Dane County is not a pure non-expansion county. Wisconsin expanded its Medicaid program’s eligibility to 100 percent of the federal poverty level for nondisabled adults without dependents at the roughly the same time as the ACA Medicaid expansion. Thus Wisconsin had likely provided financial access to behavioral health services to the population at highest risk for criminal justice involvement. This may attenuate differences in outcomes between the Midwest pair.

### Southwest

In the Southwest, we deliberately chose counties on the U.S.-Mexico border with large Hispanic-Latino populations—83 percent in El Paso and 36 percent in Pima (U.S. Census Bureau 2015). An estimated 77 percent of El Paso’s population had graduated from high school by age twenty-five versus 88 percent in Pima County (U.S. Census Bureau 2011). The proportion of the population age eighteen or younger in El Paso was 27 percent to 21 percent in Pima. Before expansion, El Paso had a poverty rate of 20 percent and median household income of $41,637, and Pima of 19 percent and $46,162, respectively (U.S. Census Bureau 2011). In 2013, El Paso had a jail rate of 324 per hundred thousand people to Pima’s 289 (Vera Institute of Justice 2018).

The two counties also have similar levels of coverage for behavioral health conditions in the Medicaid program (see table A1). However, Texas limits the number of individual and group therapy sessions to thirty per person per year, whereas Arizona has no limit. Additionally, Texas does not provide coverage for residential psychiatric treatment, and Arizona does.

In terms of diversion and integration programs, El Paso passed the Criminal Justice Mental Health Jail Diversion Collaboration Resolution in 2011. The purpose of this resolution was to engage community stakeholders in efforts to divert both prearrest and postarrest individuals with behavioral health conditions, an estimated 30 to 35 percent of the jailed population, from the justice system to appropriate treatment.

The sheriff in Pima County also implemented initiatives to reduce criminal justice involvement among individuals with behavioral health conditions in 2011. At this time, Pima County built a Crisis Response Center and Behavioral Health Pavilion to provide integrated care to those experiencing behavioral health crises and help them avoid unnecessary incarceration. Additionally, the Pima County Sheriff’s Department Mental Health Investigative Support Team coordinates responses with Pima County Behavioral Health and other law enforcement agencies when individuals with behavioral health conditions are involved in criminal justice events.

### Southeast

In the Southeast, Hinds County and East Baton Rouge had large African American populations—47 percent in East Baton Rouge and 72 percent in Hinds County before expansion (U.S. Census Bureau 2015). More than 22 percent of East Baton Rouge’s population was under the age of eighteen in 2015, versus 25 percent in Hinds County (U.S. Census Bureau 2015). More than 89 percent of the East Baton Rouge population had graduated from high school by age twenty-five, relative to nearly 86 percent in Hinds County (U.S. Census Bureau 2011). Both counties had pretrial jail rates well above the national average (327 per hundred thousand)—466 in Hinds and 537 in East Baton Rouge in 2015 (Vera Institute of Justice 2018). The poverty rates were 27 percent and 19 percent in Hinds and East Baton Rouge, respectively, and median household income was $37,324 in Hinds and $49,285 in East Baton Rouge prior to expansion (U.S. Census Bureau 2011).

Mississippi and Louisiana provide similar levels of coverage for behavioral health services, and neither state covers residential psychiatric treatment (see table A1). Additionally, Louisiana does not cover methadone for the treatment of opioid use disorder, though Mississippi does.

Louisiana has expanded its Medicaid program under the ACA, but little progress has been made in engaging local sheriffs to enroll eligible jail inmates in the state’s expanded Medicaid program, even as part of reentry planning. In addition to these challenges, efforts at implementing a program to facilitate Medicaid enrollment upon discharge have been stymied by the East Baton Rouge’s data system, which maintains information not by Social Security number but instead by an inmate ID unique to the jail.

Similar obstacles exist in Hinds County, Mississippi. The county is under a U.S. Department of Justice consent decree for failing to provide adequate health-care services in its jail system. In addition, little capacity exists in the community behavioral health-care system to treat people leaving jail, and reentry planning is severely limited. In fact, in 2015, a newly elected sheriff terminated a mental health diversion program, which demonstrated savings to the jail system in its first year of implementation.

These statistics and descriptions suggest a number of similarities between the counties in each county pair in terms of the nature of their local populations, the economic circumstances in each county, the likely pressures on the law enforcement system and efforts (or lack of them) to integrate the criminal justice and behavioral health-care systems. The qualitative data support our inferences within each county pair. However, we have also identified circumstances where changes to the state’s Medicaid program (Dane County, Wisconsin) or criminal justice system practices (Hinds County, Mississippi) may drive our results toward finding no changes.

### Description of Data

Data were obtained from each county jail and include person-level booking and release dates. Arrestees in each county are given unique identification numbers upon first arrest in the county and thus could be followed over the study period. The data spanned four years in each county—two before and two after Medicaid expansion. Additionally, each county pair provided individual-level characteristics of arrestees, but these characteristics differed across counties. Each county pair provided at least the gender of the arrestee. In the Southwest and Southeast counties, the county provided the race-ethnicity of the arrestee. In the Southwest and Midwest counties, the dataset contained a measure of the severity of the crime. In the Southwest, we know whether the arrest was for a technical violation; and in the Midwest, we know whether the arrest was for a misdemeanor, felony, or some other charge.

Using these data, we created three distinct periods over the forty-eight months of data—a lookback period, a preexpansion observation period, and a postexpansion observation period. The six-month lookback captures a person’s prior history with the criminal justice system. This period was followed by eighteen months of the preexpansion and twenty-four months of the postexpansion period. Arizona and Minnesota expanded their Medicaid programs beginning January 1, 2014; Louisiana did so beginning July 1, 2016. Observations are at the person-month level.

### Cohort Construction and Outcome Measures

Within each county, people entered the study cohort when first arrested and were followed throughout the study. If someone is arrested in the preexpansion period, we considered them at risk for the remainder of the study. If someone is arrested only in the postexpansion period, we considered them at risk for the remainder of that period only. Those who were arrested only in the lookback period were excluded from the analysis. Arrestees who were booked into the county jail as a transfer from one prison to another (either state or federal) or as a hold for state or federal charges were also excluded.

Most of the literature on the relationship between Medicaid coverage or behavioral health services and recidivism relies on a single outcome—rearrest. Evidence from Florida suggests that 30 percent of men and 20 percent of women are rearrested within eighteen months of being released (Costopoulos et al. 2017). Relying on this single measure may miss other ways in which access to behavioral health services may affect recidivism, such as reducing the frequency of interactions with the criminal justice system. We therefore not only focus on rearrest rates, but also include the number of arrests in our study.

### Quasi-Experimental Design

Despite the high rate of Medicaid eligibility in the jailed population, our data do not indicate who was eligible for or enrolled in Medicaid coverage after expansion or who received behavioral health services as a result of increased financial access to these services. Therefore, in an intent-to-treat analysis, we compare our outcomes of interest before and after Medicaid expansion between expansion and non-expansion counties for each county pair. Evidence is strong, however, on the likely eligibility for Medicaid of the reentry population in expansion states. The GAO estimated that for two states that expanded Medicaid (New York and Colorado), 80 to 90 percent of people in their prison systems were eligible for Medicaid in 2014 (GAO 2014). Similarly, Massachusetts reports that 91 percent of those released from its correction system were eligible for Medicaid.

We conducted comparative interrupted time series (CITS) regression analysis. The CITS design takes advantage of an exogenous source of variation (the state’s decision to expand Medicaid) between a treatment and comparison group (Lopez Bernal, Cummins, and Gasparrini 2018; St. Clair, Cook, and Hallberg 2014; Shadish, Cook, and Campbell 2002) and allows for the estimation of both short-and longer-term relationships between the outcome and exposure.

In both a difference-in-differences and CITS design, the counterfactual is constructed by assuming the change seen in the comparison group from before Medicaid expansion to after Medicaid expansion would be the same change seen in the treated group if not for the treatment. However, in a difference-in-differences design, the assumption is constrained so that the average change between the two groups is the same. In CITS, the counterfactual is constructed by assuming that the change in the level and trend from the linear extrapolation of outcomes before expansion in the comparison group is a good stand-in for the unobserved outcomes in the treated group. The only reason for differential deviation from these linear trends is the interruption in the treated group, that is, all other reasons for deviation affect the treated and control group in the same way. Given the drivers of the outcome in this study (policing practices, criminal justice practices, and access to behavioral health services) and how they may vary between the counties, assuming linearity in the time trend of the outcomes in each of the two groups seems more reasonable than assuming that they change in the same average way over time.

### Statistical Analysis

Because of heterogeneity in the criminal justice and health-care delivery systems, legal dynamics (such as border policy), and populations across our study pairs, we chose to analyze each county pair separately. First, we computed preexpansion means and variances for the probability of rearrest and number of arrests in both the treatment and comparison counties for the full sample and for stratified samples in each county pair. Because the policing, criminal justice, behavioral health, and health insurance systems treat people differently based on observed gender and race-ethnicity, stratification on these dimensions aims to ensure that we are making apples-to-apples comparisons in our estimation strategy. Next, we computed preexpansion monthly means of each outcome of interest to create preexpansion trends.

If Medicaid expansion leads to reduced probability of rearrest and number of arrests, then the composition of the pre-and postexpansion cohorts may differ in each observation period, with people arrested in the latter period being at higher risk, on average, than those arrested in the first period. We thus computed the number of individuals arrested and the number of arrests in both periods to check for compositional shifts in severity.

For the probability of rearrest, we used a linear probability model.^3^ For the number of arrests (a count), we used ordinary least squares (OLS).^4^ We used the following general specification for each of the outcomes:

$$y_{ist}=\alpha +\beta_{1}X_{i}+\beta_{2}X_{i}TIME_{t}+\beta_{3}P_{it}+\beta_{4}P_{it}TIME_{t}\;+\lambda_{1}\text{TREA}T_{s}+\lambda_{2}POST_{t}+\lambda_{3}\text{TREN}D_{st}\;+\lambda_{4}\text{TREA}T_{s}*\text{TREN}D_{st}+\lambda_{5}\text{TREN}D_{st}*POST_{t}\;+\gamma \;\text{TREA}T_{s}*Post_{t}+\eta \text{TREA}T_{S}*POST_{t}*\text{TREN}D_{st}+\text{QUARTE}R_{t}+\varepsilon_{ist},$$

where *i* is an individual in the cohort, *s* is the county, and *t* is month. ***X***_*i*_ is a vector of time-invariant individual characteristics that are allowed to vary with the outcome over time, ***P***_*it*_ is a vector of time-varying individual characteristics with effect, *γ* is the CITS estimator for the level shift in the outcome, *η* is the CITS estimator for the trend shift in the outcome and the parameter of interest, QUARTER is a vector of quarter fixed effects.

Time and individual-varying characteristics consist of charge severity—whether the arrest was a misdemeanor or felony (Midwest) or whether for a technical violation (Southwest). Time-invariant, individual-varying characteristics include the arrestee’s gender, race-ethnicity (where available), and prior history with the criminal justice system. We interacted both the time-varying and time-invariant person-level covariates with the monthly time trend to adjust for the possibility that the relationship between the covariates and the outcome of interest is also time varying. Within each county pair, we used a Bonferroni correction for multiple testing.

As noted, we stratify the CITS regression analyses in each county pair by gender and major local racial-ethnic groups. If baseline differences in racial or ethnic make-up were driving both arrest patterns and Medicaid expansion, then we would expect the causal estimates for these minority groups to differ significantly from that of the pooled sample.

### Robustness Checks and Falsification Tests

A falsification test often used in CITS artificially places the empirical implementation date within a clean study period (that is, either the before or the after period). It could be that the outcome is noisy and that estimated effects might appear by chance. We varied the implementation date to three months in the preexpansion period—months seven, ten, and thirteen in the time series. Varying the implementation date within only the preexpansion period is preferable to strategies that vary it within the study period as a whole, because the full study includes the treatment effect and may lead to detecting a spurious effect at a falsified intervention point. Our falsification strategy is also not ideal because it cannot account for any anticipatory effects of Medicaid expansion—particularly in the Southeast county pair, where other coverage provisions of the ACA had been in place for more than two years before expansion. Thus we expect a weakening of the estimated impacts as the intervention date is moved away from the true date.

Additionally, because the before and after observation windows are of different length, arrestees are at risk for being arrested longer in the postexpansion period than in the preexpansion. This difference should be handled by the CITS design because the observation periods are shared by the intervention and comparison groups. Nonetheless, we checked for any effect of this discrepancy by conducting sensitivity analyses where we truncate the post-period to eighteen months to balance the exposure time before and after.

Finally, case studies raise general concerns about the validity of statistical inference. Given only two clusters, estimating and accounting for intracluster correlation is not possible (both the within and between-group variance cannot be estimated in only two groups). Thus typical econometric procedures for standard error adjustment (such as clustering, bootstrapping, permutation inference) are not feasible in this setting. We conducted the full sample CITS at the county-month year as a check on statistical inference. Aggregating to the cluster-time level results in less biased standard errors even in a small number of clusters (Rokicki et al. 2018) but prevents us from adjusting for individual-level covariates.

## RESULTS

In the preexpansion period, the three Medicaid expansion counties arrested more people than the comparison counties (22,146 versus 9,489 in the Midwest pair; 32,222 versus 26,576 in the Southwest pair; and 19,185 versus 7,639 in the Southeast pair), because the populations of the expansion counties are larger than those of the non-expansion counties. Indeed, the proportion of the total population arrested preexpansion is similar in expansion and non-expansion counties—1.6 percent of each county in the Midwest, 3.2 percent of both counties in the Southwest, and 3.6 and 2.6 percent respectively in the Southeast. Similarly, the number of arrests are higher in the Medicaid expansion counties prior to expansion than in the non-expansion counties (33,082 versus 13,405 in the Midwest, 46,569 versus 31,966 in the Southwest, and 22,905 versus 9,499 in the Southeast).

In the postexpansion period, the number of people arrested was higher in both the expansion and non-expansion counties of each pair (26,759 and 11,606 in the Midwest, 38,186 and 31,300 in the Southwest, and 20,371 and 9,300 in the Southeast) and arrests were also more numerous (42,904 versus 17,758 in the Midwest, 59,343 versus 40,924 in the Southwest, and 25,311 versus 12,166 in the Southeast). This suggests that the composition of the cohort may be changing from the before to the after period.

A primary concern with compositional shifts leading to fewer arrests is the possible implication that the least risky individuals (those with fewer impairing conditions) would be disproportionately enrolled in Medicaid and have greater financial access to behavioral health-care services after expansion because they had the cognitive and functional capacity to do so. If individuals with less severe conditions were more likely to enroll, our cohort would include a larger proportion of people more likely to have higher rates of recidivism and commit potentially more serious crimes. This scenario would lead to bias and overestimation of the effect of expansion on recidivist behavior. However, the direction of the observed change (an increase in the number of individuals arrested) implies that the marginal individual arrested in the postexpansion period is likely not of higher risk than that in the preexpansion period.

Data on the types of arrests supports this interpretation. Of the arrests in the Southwest county pair, 3,933 and 3,394 were for a parole violation in the before and after periods, respectively. Additionally, 22,951 (49.4 percent) of the Midwest arrests in the preexpansion period were for misdemeanors and 17,036 (50 percent) in the postexpansion period; 15,121 (32.5 percent) and 11,191 (32.8 percent) were for felonies. Arrest composition was almost identical, suggesting that the marginal individuals arrested after expansion are at roughly the same risk as those arrested before expansion.

### Cohort and Arrest-Level Characteristics

In general, arrestees were much more likely to be men in each county pair (see table 1). The proportion of the cohort that is female was greater in the non-expansion county (Dane, Wisconsin) in the Midwest pair (24.2 to 22.7 percent), in the non-expansion county (El Paso, Texas) in the Southwest pair (27.5 to 26.1 percent), and in the expansion county (East Baton Rouge Parish, Louisiana) in the Southeast pair (24.9 to 17.7 percent). These differences are small in all except the Southeast pair. In terms of racial composition, the proportion of Hispanic-Latino arrestees in the preexpansion period in El Paso County was almost twice that in Pima County, Arizona (81.6 to 41.7 percent). Similarly, arrestees in the preexpansion period in Hinds County, Mississippi, were more likely to be black than those in East Baton Rouge (80.8 to 66.1 percent).

In the Southwest county pair, a preexpansion period arrest in Pima County was nearly five times more likely to be for a parole violation than an arrest in El Paso County (7.4 versus 1.5 percent of all arrests). Similar arrests in Dane County, Wisconsin, were more likely than those in Hennepin County, Minnesota, to be for misdemeanors (55.9 to 46.7 percent) or felonies (37.3 to 30.6 percent). Although these baseline differences in arrestee demographics and arrest charge or severity were stable across the study period, they suggest that our comparison counties differ, at least in racial-ethnic composition. We stratified regression analyses by gender and dominant minority group to partially address within-pair county heterogeneity.

**Figure 2.**
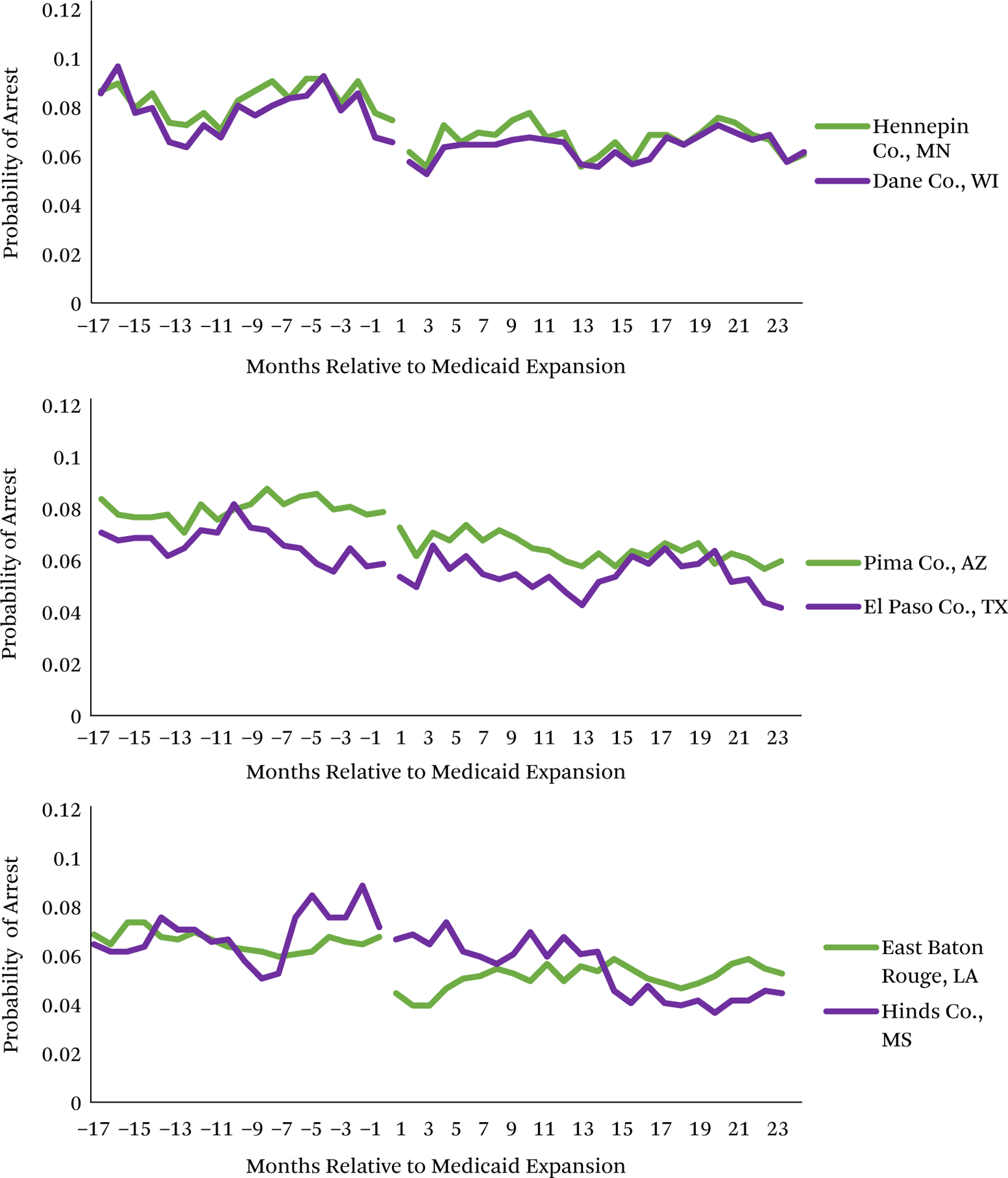
Probability of Arrest in Each County over the Study Period *Source:* Authors’ analysis of arrest data from six county jails. *Note:* In the Midwest and Southwest, the pre-period observation window is July 1, 2012, through September 30, 2013. In the Southeast, the pre-period observation window is January 1, 2015, through March 31, 2016. In the Midwest and Southwest, the post-period observation window is July 1, 2014, through December 31, 2015. In the Southeast, the post-period observation window is January 1, 2017, through June 30, 2018. Medicaid expansion in the Midwest and Southwest county pairs occurred on January 1, 2014. Medicaid expansion in the Southeast county pair occurred on June 1, 2016.

Figure 2 presents the trends for the likelihood of being arrested in each month relative to Medicaid expansion. The trends before and since expansion in the Midwest county pair appeared to be fairly stable and are not changing in level or trend over time. Despite possible level shifts, the unadjusted monthly trends in the Southwest and Southeast county pairs did not appear to change much after expansion. However, visualizations of the raw outcomes are not particularly helpful in assessing a treatment effect, given that estimation is done based on the residuals rather than the raw data (Bilinski and Hatfield 2018).

### Probability of Rearrest

The probability of rearrest in the preexpansion period was higher in Hennepin County, Minnesota (expansion; 28.2 percent; 95% CI: 27.6, 28.9) than in Dane County, Wisconsin (non-expansion; 26.6 percent; 95% CI: 25.7, 27.6) and in Hinds County, Mississippi (non-expansion; 20.2 percent; 95% CI: 19.3, 21.1) than in East Baton Rouge, Louisiana (expansion; 16.0; 95% CI: 15.5, 16.5) (see table 2). The greatest difference in the probability of rearrest was in the Southwest county pair, where the Pima County, Arizona (expansion) rate was 11.5 percentage points higher than that of El Paso County, Texas (non-expansion).

When stratified by gender and race-ethnicity in each county pair, the pattern of results was the same. The probability of rearrest among male and female arrestees was higher in Hennepin County, Pima County, and Hinds County. Among black arrestees in the Southeast, the probability of rearrest was 3.4 percentage points higher in Hinds County than in East Baton Rouge. Additionally, the probability of rearrest among Hispanic-Latino individuals in the preexpansion period was 8.1 percentage points higher in Pima County than in El Paso County. Although differences in the preexpansion period trend may be cause for concern in a difference-in-differences analysis, baseline or trend differences in the outcomes do not invalidate causal inferences for CITS, which models differential levels and trends in both the pre-to the postexpansion period.

**Figure 3.**
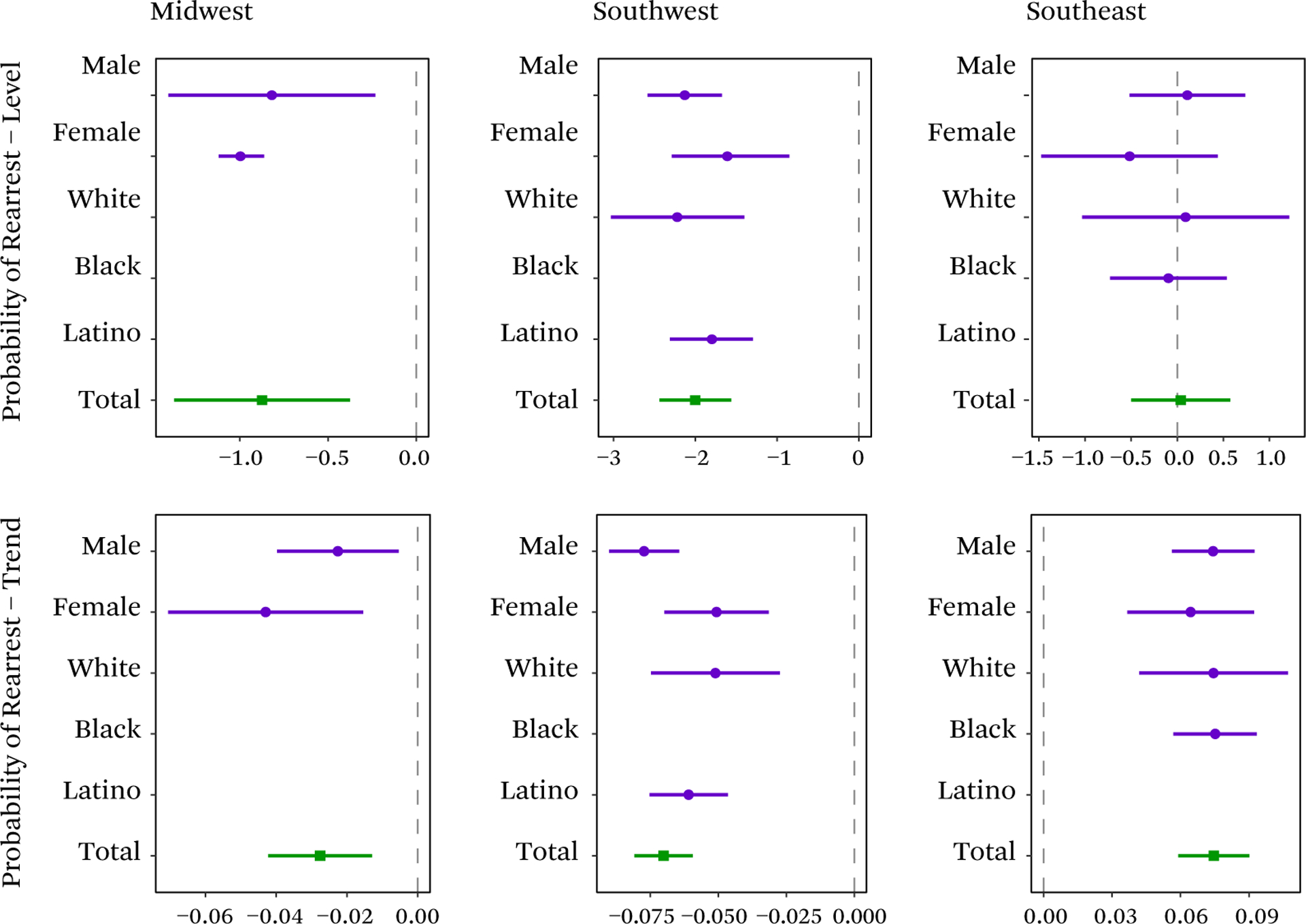
Changes in the Probability of Rearrest Between Medicaid Expansion and Non-Expansion Counties *Source:* Authors’ analyses of arrest data from county jails. *Note:* The study period for the Midwest and Southwest is from July 1, 2012, through December 31, 2015. The pre-expansion period for the Southeast is from January 1, 2015, through June 30, 2018. Observations are at the person-month level. Estimates are from comparative interrupted time series regressions. Regressions are linear probability models. Each full sample regression is adjusted with gender and prior contact with the criminal justice system. The Midwest pair also adjusts for whether the arrest was a felony or misdemeanor and the interaction of this variable with the monthly counter. The Southwest county pair also adjusts for whether the arrest was for a parole violation and for whether the arrestee was Hispanic-Latino plus the interactions of these two variables with the monthly counter. Regressions using the Southeast county pair also adjust for whether the arrestee was African American and the interaction of this variable with the monthly time trend. Stratified regression analyses in each county pair adjust for these same covariates except for the variable that the sample was stratified on.

After Medicaid expansion, the short-term probability of rearrest (the level change) declined by a statistically significant amount in the Midwest and Southwest county pair (see figure 3). The largest decline was in the Southwest county pair, for which the probability of being rearrested declined by 2.0 percentage points (95% CI: −2.4, −1.6) in the month following expansion, which is a 7.27 percent decrease from the preexpansion period mean. The estimated effect of Medicaid expansion on rates of rearrest in Pima County relative to El Paso County was greatest among white (−2.2 percentage points in the month after expansion; 95% CI: −3.0, −1.4) and male arrestees (−2.1 percentage points; 95% CI: −2.6, −1.7). Reductions in recidivism were similar among female and Hispanic-Latino arrestees relative to the full sample.

In the Midwest county pair, the probability of an individual being rearrested declined by 0.87 percentage points in the month after expansion (95% CI: −1.4, −0.4) in Hennepin County relative to Dane County, which amounts to a 2.9 percent decrease in the probability of rearrest. Similar declines were seen among male and female arrestees. In the Southeast county pair, Medicaid expansion was not associated with changes in the probability of rearrest.

We also found sustained decreased rates of recidivism per month for arrestees in the Midwest (−0.03 percentage points; 95% CI: −0.04, −0.01) and Southwest (−0.07 percentage points; 95% CI: −0.06, −0.08) county pairs (see figure 3). The effects were similar for all subgroups examined in these two county pairs. Despite no change in the level of the outcome in the Southeast, arrestees in East Baton Rouge experienced a change in trend relative to their counterparts in Hinds County (0.07 percentage points; 95% CI: 0.06, 0.09). This change is similar for male, female, and African American arrestees.

By extrapolating the level and trend change to the end of the study period, we found that Medicaid expansion led to an average decline in the probability of rearrest of 1.49 percentage points (an 4.92 percent decrease) in Hennepin County relative to Dane County and of 3.6 percentage points (13.1 percent) in Pima County relative to El Paso County. In the Southeast, a differential trend increase resulted in a 1.61 percentage point increase (10.1 percent) in East Baton Rouge relative to Hinds County.

### Number of Arrests

Like the descriptive statistics for the probability of rearrest, the average number of arrests in the preexpansion period was higher in Hennepin County, Minnesota, than in Dane County, Wisconsin (1.54 to 1.46). The average number of arrests for men and women in the expansion county was also higher than that of the non-expansion county in the Midwest pair (see table 2). Similarly, in the full sample and among all stratifications, the average number of arrests was higher in Pima County, Arizona, than in El Paso County, Texas (1.50 to 1.22). Arrestees in Hinds County, Mississippi (non-expansion) had a higher average number of arrests than those in East Baton Rouge, Louisiana (1.27 to 1.21), which was also the case for male, female, and black arrestees in the Southeast pair.

**Figure 4.**
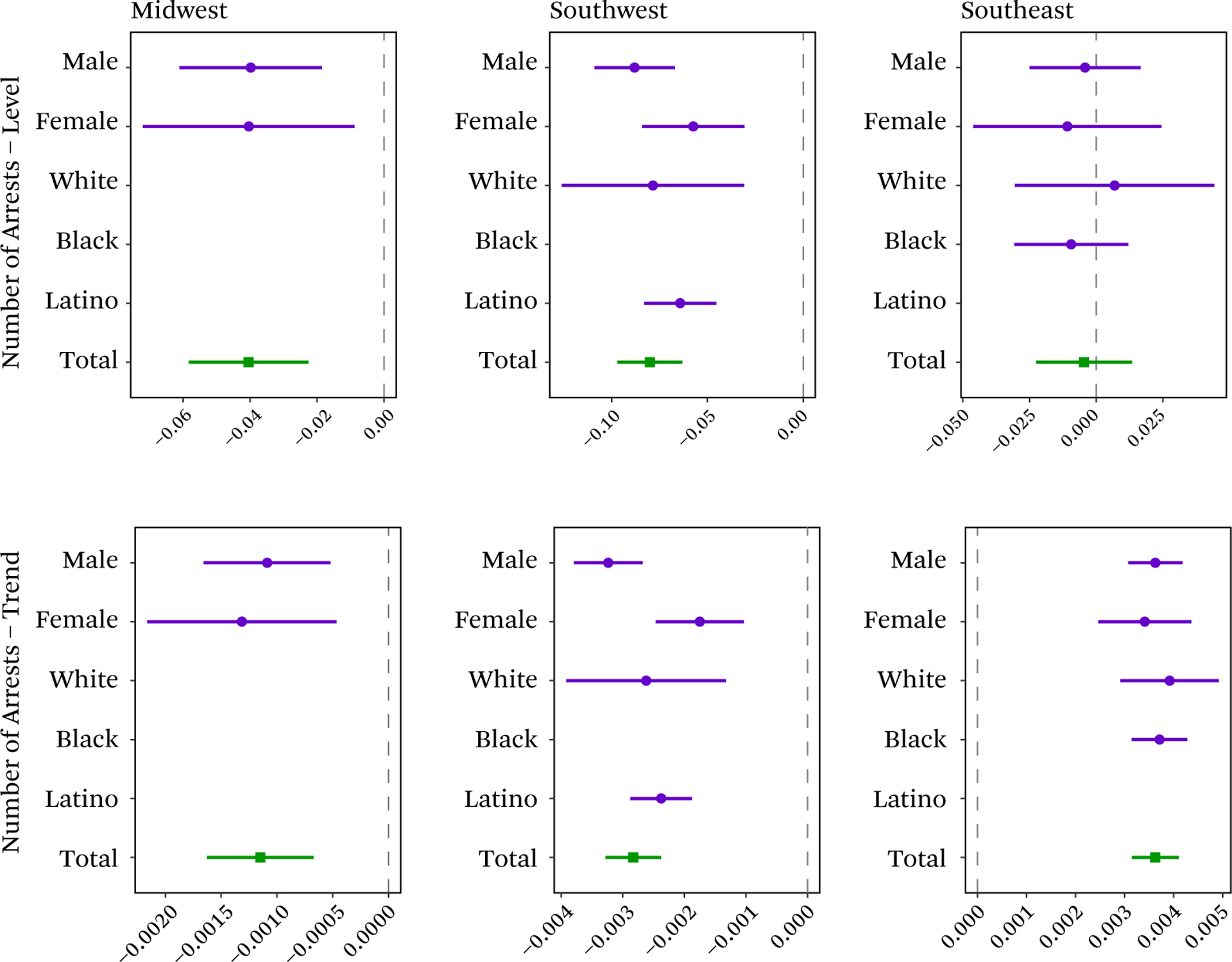
Changes in the Number of Rearrests Between Medicaid Expansion and Non-Expansion Counties *Source:* Authors’ analyses of arrest data from county jails. *Note:* The pre-expansion period for the Midwest and Southwest is from July 1, 2012, through December 31, 2015. The pre-expansion period for the Southeast is from January 1, 2015, through June 30, 2018. Observations are at the person-month level. Estimates are from comparative interrupted time series regressions. Each full sample regression is adjusted with gender and prior contact with the criminal justice system. The Midwest pair also adjusts for whether the arrest was a felony or misdemeanor and the interaction of this variable with the monthly counter. The Southwest county pair also adjusts for whether the arrest was for a parole violation and for whether the arrestee was Hispanic-Latino plus the interactions of these two variables with the monthly counter. Regressions using the Southeast county pair also adjust for whether the arrestee was African-American and the interaction of this variable with the monthly time trend. Stratified regression analyses in each county pair adjust for these same covariates except for the variable that the sample was stratified on.

Overall, Medicaid expansion resulted in a decline in the average number of arrests per person in the Midwest (−0.04; 95% CI: −0.02, −0.06) and Southwest (−0.08; 95% CI: −0.06, −0.10) expansion counties relative to the respective non-expansion counties in the month after Medicaid expansion (see figure 4). In both the Midwest and the Southwest, the change in the number of arrests did not differ significantly in the stratified samples relative to the full sample estimate.

Much as in rearrests, the level change in the number of arrests in the Southeast county pair is close to zero and not statistically significant (−0.004; 95% CI: −0.02, 0.01), suggesting the lack of any immediate impact of Medicaid expansion and recidivism.

The longer-term effects of Medicaid expansion on the average number of arrests per person were similar to the pattern in the rearrest analyses. The average number of arrests per person per month decreased more in Hennepin County than in Dane County (−0.001; 95% CI: −0.001, −0.002). The average number decreased for the full sample, male, female, and Hispanic-Latino arrestees (roughly 0.003 per month) in Pima County relative to El Paso County. The average number after Medicaid expansion increased for all groups in East Baton Rouge relative to Hinds County (0.004 arrests per month; 95% CI: 0.003, 0.004).

Via linear extrapolation of the level and trend change, we found that Medicaid expansion led to an average decline of 0.1 arrests per person in Hennepin County relative to Dane County, and an average decline of 0.2 arrests in Pima County over El Paso County two years after Medicaid expansion (the end of the study period). In terms of relative changes, this is a 5.8 percent decrease in the Midwest and a 13.3 percent decrease in the Southwest. Taking into account only the trend increases in the Southeast, Medicaid expansion resulted in 0.2 more arrests per person in East Baton Rouge than in Hinds County, a 12.2 percent increase.

### Falsification and Sensitivity Analyses

As suggested, falsification tests such as these may not be ideal when assessing whether we are isolating the causal effect of Medicaid expansion on recidivist behavior. These tests did not permit specifying any ramp up to Medicaid expansion, particularly in the Southeast, where the ACA’s other coverage provisions had been in place for more than two years before Medicaid expansion. With these caveats, the falsification tests suggested that we were capturing the causal effect (see tables A2, A3).

In all three county pairs, no change was detected in the level at the false implementation points for the probability of rearrest or the number of arrests. However, a change in the slope of the line was apparent just prior to expansion for both outcomes in the Midwest and Southwest.

In addition to these falsification tests, we also conducted sensitivity analyses. When we truncate the postexpansion period to be of equal length to the preexpansion period, we find no real differences in our estimates, with the exception of the Southeast county pair (see table A4). In the Southeast, the level estimate for both outcomes is significantly higher at eighteen months than twenty-four months postexpansion. When we collapse our analyses to the county-month level instead of the person-month level, we find that our standard errors increase in each county pair and for both outcomes (see table A5). However, this does not change the interpretation of statistical significance, except in the level and trend estimates for the probability of rearrest in the Midwest pair.

### Limitations

The study has several limitations. First, data consisted of booking data. We did not know who gains health insurance under Medicaid expansion and therefore conducted an intent-to-treat analysis. Additionally, we could not differentiate between individuals who are lost to follow up and those who are never rearrested. We assumed that rates of attrition are similar over time and across the counties in each county pair.

Second, from interviews and site visits, we identified changes to the behavioral health and criminal justice systems that add context to the results, but also highlighted that in some cases, changes may not be attributed entirely to Medicaid expansion. Hinds County, Mississippi, discontinued a mental health diversion program in late 2015 after the current sheriff was elected in August 2015 (expansion happened in Louisiana in July 2016), despite evidence that the program saved the county $250,000 in the year after implementation.

Third, baseline differences in the composition of each county’s arrested population and the overall arrest activities may suggest that these are not perfect comparison counties. We chose the counties based on their similarity on the county’s full demographic characteristics, rather than the characteristics of the jailed population. To the extent that the characteristics of the jailed populations and differential policing and arrest activity were stable over time, our design netted out these differences.

## DISCUSSION

Overall, Medicaid expansion reduced both the probability of rearrest and the number of arrests in two of the three county pairs. In the Midwest and Southwest, the estimated effects at two years after expansion were consistent with estimates from other studies on the relationship between access to health-care services and recidivism (between a 5 and 13 percent decrease). Additionally, the mixed nature of the findings (an increase in the Southeast) is also consistent with prior literature.

These estimates are similar to other initiatives to reduce recidivism. Adult drug courts reduce recidivism rates by roughly 8 percent (Aos, Miller, and Drake 2006). One meta-analysis of educational and vocational training programs found that these programs were associated with a 13 percentage point decrease is recidivism (Davis et al. 2013); another estimated these programs to reduce recidivism by 7 to 9 percent (Aos, Miller, and Drake 2006). However, many of the studies included in these meta-analyses have the same selection issues as in previous studies on the effect of increased access to health-care services on recidivism.

However, our estimates might be smaller than the true effect of Medicaid expansion on recidivism. We did not measure first-order effects—health insurance coverage and access to care—of Medicaid expansion in the jail-involved population; nor did we measure the change in recidivism in individuals who obtained Medicaid coverage and subsequent behavioral health treatment. If we were able to conduct a treatment-on-the-treated analysis, then our estimates would scale by the proportion of individuals who enrolled in Medicaid coverage due to expansion. If we were to use the proportion of jail-involved individuals eligible for Medicaid expansion from Marsha Regenstein and Sara Rosenbaum (2014), scaling our estimates would suggest that expansion is associated with a 16 to 32 percent reduction in the rate of recidivism.

In the Southeast, we failed to detect a change in the level in East Baton Rouge relative to Hinds County in the implementation of Medicaid expansion, and the change in the slope resulted in an overall increase in the probability of rearrest and number of arrests twenty-four months after expansion. This could be a result of changes to behavioral health and criminal justice practices required by the federal consent decree in Hinds County and the lack of integration and coordination between these two systems in East Baton Rouge Parish.

Additionally, we stratified our analyses by race and gender to address within-pair heterogeneity and make comparisons across individuals who are treated more similarly by the policing, health-care, and criminal justice systems. The estimates for these stratified groups were either the same size or larger than the full sample, which strengthens our inferences. Moreover, our qualitative analysis of the efforts occurring in each of the counties allowed us to draw out the contexts that may make Medicaid expansion more or less effective in reducing recidivist behavior. Indeed, our results mirrored the previous literature—enhanced financial access to health-care services contributes to a reduction in recidivism.

Other contributing factors include coverage of evidence-based treatment for mental illnesses and substance use disorders; adequate capacity in the community’s behavioral health treatment system; the provision of mental illness and substance use disorder treatment services in jail, both before adjudication and while incarcerated; the coordination and continuity of care across the criminal justice and behavioral health-care systems; the implementation of jail diversion programs that keep individuals with mental illness or substance use disorder from entering the criminal justice system; and the availability of other social programs, such as supportive housing and employment, that improve the social status of individuals with mental illness or substance use disorder. Reducing rates of rearrest, particularly for individuals with severe mental illness or substance use disorders, requires coordinated efforts between multiple social service systems and increased integration of those systems could be an important policy lever to increase time in the community.

## Supplementary Material

Supplement

## Figures and Tables

**Table 1.** Comparison of County-Level General Population Characteristics and Pre-Expansion Sample Characteristics

	Midwest		Southwest		Southeast	
	Hennepin	Dane	Pima	El Paso	EBRP	Hinds
	N = 22,146	N = 9,489	N = 32,222	N = 26,576	N = 19,185	N = 7,639
**General population**						
Population	1,223,149	523,643	1,010,025	835,593	446,753	242,891
Younger than eighteen	22.2	21.0	23.0	27.9	22.8	25.0
White	75.6	85.8	53.0	13.1	48.8	25.8
High school diploma or equivalent	92.6	95.0	87.6	75.7	89.4	85.8
Median household income	$65,834	$62,865	$46,162	$41,637	$49,285	$37,324
Pretrial jail rate (per hundred thousand)	82	122	289	324	537	466
**Jail-involved population**						
Percent Female	22.7 (22.6–22.8)	24.2 (24.0–24.4)	26.1 (26.0–26.2)	27.5 (27.4–27.6)	24.9 (24.8–25.1)	17.7 (17.5–17.9)
Percent black/African American	–	–	–	–	66.1 (66.0–66.3)	80.8 (80.6–81.0)
Percent Hispanic/Latino	–	–	41.7 (41.6–41.8)	81.6 (81.5–81.7)	–	–
**Arrest characteristics**						
Parole violation	–	–	7.4 (7.2–7.6)	1.5 (1.4–1.6)	–	–
Misdemeanor	46.7 (46.2–47.3)	55.9 (55.1–56.8)	–	–	–	–
Felony	30.6 (30.1–31.1)	37.3 (36.5–38.1)	–	–	–	–

*Source:* General population data come from the American Community Survey (U.S. Census Bureau 2011, 2015). Pretrial jail rate comes from Vera Institute of Justice 2018.

*Note:* Numbers in percentages unless otherwise indicated. Values are means and 95% confidence intervals. The proportion of individuals younger than eighteen and the proportion of individuals who are white are 2015 one-year estimates. The proportion who have a high school diploma or equivalent by age fifteen, the median household income, and the percent living in poverty are 2015 five-year estimates. The population-standardized prejail incarceration rate is for 2013 and comes from the Vera Institute’s trends on jail. Jail-involved population comes from authors’ analyses of arrest data from county jails. Sample sizes represent the preperiod number of individuals arrested in each county. The preexpansion period for the Midwest and Southwest is from July 1, 2012, through December 31, 2013. The preexpansion period for the Southeast is from January 1, 2015, through June 30, 2016.

**Table 2.** Pre-Medicaid Expansion Outcomes for Full Sample and Stratified Sample

	Midwest		Southwest		Southeast	
	Hennepin	Dane	Pima	El Paso	EBRP	Hinds
	N = 22,146	N = 9,489	N = 32,222	N = 26,576	N = 19,185	N = 7,639
**Probability of rearrest**						
Full sample	30.2 (29.6–30.8)	28.4 (27.5–29.3)	27.5 (27.0–28.0)	16.0 (15.6–16.5)	16.0 (15.5–16.5)	20.2 (19.3–21.1)
Male	31.9 (31.2–32.6)	30.0 (29.0–31.1)	29.0 (28.4–29.6)	17.0 (16.5–17.5)	17.3 (16.6–17.9)	21.6 (20.6–22.7)
Female	24.7 (23.5–25.9)	23.5 (21.8–25.2)	23.3 (22.4–24.2)	13.4 (12.6–14.2)	12.2 (11.3–13.1)	13.7 (11.8–15.5)
Black	–	–	–	–	17.5 (16.8–18.2)	20.9 (19.9–22.0)
Hispanic-Latino	–	–	26.1 (25.4–26.9)	16.0 (15.5–16.5)	–	–
**Number of arrests**						
Full sample	1.54 (1.52–1.55)	1.46 (1.45–1.48)	1.50 (1.49–1.51)	1.22 (1.21–1.23)	1.21 (1.20–1.22)	1.27 (1.26–1.28)
Male	1.57 (1.56–1.59)	1.50 (1.47–1.52)	1.54 (1.52–1.56)	1.23 (1.22–1.24)	1.23 (1.22–1.24)	1.29 (1.27–1.31)
Female	1.42 (1.39–1.44)	1.36 (1.33–1.39)	1.40 (1.38–1.42)	1.18 (1.17–1.20)	1.15 (1.14–1.16)	1.17 (1.14–1.20)
Black	–	–	–	–	1.23 (1.22–1.24)	1.28 (1.26–1.30)
Hispanic-Latino	–	–	1.44 (1.42–1.45)	1.22 (1.21–1.23)	–	–

*Source:* Authors’ analyses of arrest data from county jails.

*Note:* Values are means and 95% confidence intervals. We only provide descriptive statistics for the predominant racial/ethnic group in each county pair, where available. The pre-expansion period for the Midwest and Southwest is from July 1, 2012, through December 31, 2013. The pre-expansion period for the Southeast is from January 1, 2015, through June 30, 2016. No race-ethnicity data were available in the Midwest, and no arrest-level data were available for the Southeast.

—indicates that variable was unavailable or not analyzed for the given county pair. Sample sizes are for the full sample—stratification based on gender or race-ethnicity reduces the sample size.

## Footnotes

1. *National Federation of Independent Business v. Sebelius*, 567 U.S. 519 (2012).

2. All supplemental appendix tables, designated in text with a leading A, are available via the online appendix at https://www.rsfjournal.org/content/6/2/244/tab-supplemental.

3. Although linear probability models (LPMs) are often used in CITS regressions due to the ease of interpreting an interaction coefficient in these models, LPMs are suitable only for probabilities between 0.2 and 0.8 not for tail probabilities. The literature suggests that the probability of rearrest may be somewhere between 20 and 30 percent. However, a logistic regression model, which handles tail probabilities better than an LPM, are difficult to interpret, particularly when estimating interaction effects (Karaca-Mandic, Norton, and Dowd 2012) and violates the additivity assumptions of both DID and CITS.

4. Count data can be modeled using OLS instead of a Poisson model, though the estimates may be smaller using OLS rather than a Poisson model (Hausman, Hall, and Griliches 1984).
