# Supplementary material for: Medicaid Expansion’s Spillover to the Criminal Justice System: Evidence from Six Urban Counties
Source: RSF. Author manuscript; Available in PMC 2020 Nov 30. (PMC7702715; doi:10.7758/rsf.2020.6.2.11)
Supplement: Supplement [file NIHMS1645996-supplement-Supplement.pdf]

Supplemental Appendix

Medicaid Expansion's Spillover to the Criminal Justice System: Evidence from Six Urban Counties

Carrie E. Fry MEd
Thomas G. McGuire PhD
Richard G. Frank, PhD

A1. Sample state differences in Medicaid coverage of behavioral health services

<table>
<tr><th></th><th colspan="2">Inpatient</th><th>Residential</th><th colspan="4">Outpatient</th></tr>
<tr><th></th><th>Psychiatric</th><th>Detoxification</th><th>Psychiatric</th><th>Individual therapy</th><th>Group therapy</th><th>Buprenorphine</th><th>Methadone</th></tr>
<tr><td>Midwest</td><td></td><td></td><td></td><td></td><td></td><td></td><td></td></tr>
<tr><td>MN</td><td colspan="2">Yes</td><td>Yes</td><td colspan="2">Yes</td><td colspan="2">Yes</td></tr>
<tr><td>WI</td><td colspan="2">$3/day; $75/stay</td><td>No</td><td colspan="2">$0.50 – $3 copay</td><td colspan="2">Copay of up to $12/month</td></tr>
<tr><td>Southwest</td><td></td><td></td><td></td><td></td><td></td><td></td><td></td></tr>
<tr><td>AZ</td><td></td><td>Yes</td><td>Yes</td><td>Yes</td><td>Yes</td><td>Yes</td><td>Yes</td></tr>
<tr><td>TX</td><td>15-day max</td><td>Requires prior authorization</td><td>No</td><td colspan="2">30/year</td><td>Yes</td><td>Only at OTP</td></tr>
<tr><td>Southeast</td><td></td><td></td><td></td><td></td><td></td><td></td><td></td></tr>
<tr><td>LA</td><td colspan="2">Yes</td><td>No</td><td colspan="2">Yes</td><td>$0.50 – $3 copay</td><td>No</td></tr>
<tr><td>MS</td><td colspan="2">Requires prior authorization</td><td>No</td><td>36/year</td><td>24/year</td><td colspan="2">$3 copay & prior authorization</td></tr>
</table>

SOURCES/NOTES All data comes from the Kaiser Family Foundation Medicaid Behavioral Health Services Database. Available at: https://www.kff.org/data-collection/medicaid-behavioral-health-services-database/.

A2. Falsification test of relationship between Medicaid expansion and the probability of re-arrest

	**Change in intercept**		**Change in slope**	
	Coefficient	*95% CI*	*Coefficient*	*95% CI*
**Midwest**				
Month 7	-0.71	-2.28, 0.85	-0.07	-0.13, -0.01
Month 10	-0.88	-2.59, 0.82	-0.09	-0.14, -0.00
Month 13	-0.73	-3.70, 2.23	-0.09	-0.18, -0.00
Month 18	-0.87	-0.38, -1.12	-0.03	-0.04, -0.01
**Southwest**				
Month 7	-0.91	-1.93, 0.10,	-0.07	-0.11, -0.03
Month 10	-0.35	-1.55, 0.84	-0.08	-0.12, -0.04
Month 13	-0.97	-3.09, 1.15	-0.21	-0.27, -0.15
Month 18	-2.00	-2.44, -1.56	-0.07	-0.08, -0.06
**Southeast**				
Month 7	-0.28	-1.18, 1.13	-0.02	-0.08, 0.03
Month 10	-0.03	-1.69, 1.63	-0.03	-0.09, 0.02
Month 13	-0.34	-0.70, 0.66	0.08	-0.02, 0.17
Month 18	0.04	-0.50, 0.58	0.07	0.06, 0.09

Sources/Notes: SOURCES Authors' analyses of arrest data from county jails. Observations are at the person-month level NOTES Estimates are from comparative interrupted time series regressions. Regressions for the likelihood of re-arrest are linear probability models. Regressions for the number of arrests are Poisson regression models. Each full sample regression is adjusted with gender and prior contact with the criminal justice system (in the pre-period) and an interaction between these variables and the running monthly counter to account for a time-varying relationship between the outcome and the covariates. The Midwest pair also adjusts for whether the arrest was a felony or misdemeanor and the interaction of this variable with the monthly counter. The Southwest county pair also adjusts for whether the arrest was for a parole violation and for whether the arrestee was Hispanic/Latino plus the interactions of these two variables with the monthly counter. Regressions using the Southeast county pair also adjust for whether the arrestee was African-American and the interaction of this variable with the monthly time trend. § denotes that p-value is not statistically significant after Bonferroni adjustment for multiple comparisons.

A3. Falsification test of relationship between Medicaid expansion and the number of arrests to three months prior to Medicaid expansion

	**Change in intercept**		**Change in slope**	
	Coefficient	*95% CI*	*Coefficient*	*95% CI*
**Midwest**				
Month 7	0.003	-0.05, 0.06	-0.0001	-0.002, 0.002
Month 10	-0.004	-0.06, 0.14	-0.001	-0.002, 0.001
Month 13	0.02	-0.08, 0.06	-0.0001	-0.004, 0.0003
Month 18	-0.04	-0.06, -0.02	-0.001	-0.002, -0.001
**Southwest**				
Month 7	0.03	-0.02, 0.08	0.003	0.005, 0.001
Month 10	0.04	-0.01, 0.08	0.002	0.0005, 0.004
Month 13	0.00	-0.07, 0.07	-0.004	-0.006, -0.002
Month 18	-0.08	-0.10, -0.06	-0.003	-0.003, -0.002
**Southeast**				
Month 7	0.01	-0.04, 0.07	0.0002	-0.002, 0.002
Month 10	-0.01	0.01, 0.05	-0.002	-0.004, -0.0004
Month 13	-0.07	-0.06, 0.04	0.003	0.001, 0.005
Month 18	-0.004	-0.02, 0.01	0.004	0.003, 0.004

Sources/Notes: SOURCES Authors' analyses of arrest data from county jails. Observations are at the person-month level NOTES Estimates are from comparative interrupted time series regressions. Regressions for the likelihood of re-arrest are linear probability models. Regressions for the number of arrests are Poisson regression models. Each full sample regression is adjusted with gender and prior contact with the criminal justice system (in both the pre- and post-period) and an interaction between these variables and the running monthly counter to account for a time-varying relationship between the outcome and the covariates. The Midwest pair also adjusts for whether the arrest was a felony or misdemeanor and the interaction of this variable with the monthly counter. The Southwest county pair also adjusts for whether the arrest was for a parole violation and for whether the arrestee was Hispanic/Latino plus the interactions of these two variables with the monthly counter. Regressions using the Southeast county pair also adjust for whether the arrestee was African-American and the interaction of this variable with the monthly time trend. [§] denotes that p-value is not statistically significant after Bonferroni adjustment for multiple comparisons.

A4. Comparison of estimates with full post-period (24 months) compared to truncated post-period (18 months)

	**Probability of Re-arrest**		**Number of Arrests**	
	Change in Level	*Change in Slope*	*Change in Level*	*Change in Slope*
**Midwest**				
24 months post	-0.87	-0.03	-0.04	-0.001
18 months post	-0.96	-0.01	-0.04	-0.001
**Southwest**				
24 months post	-2.00	-0.07	-0.08	-0.003
18 months post	-1.84	-0.07	-0.07	-0.003
**Southeast**				
24 months post	0.04	0.07	-0.005	0.004
18 months post	0.17	0.05	0.14	0.004

Sources/Notes: SOURCES Authors' analyses of arrest data from county jails. Observations are at the person-month level NOTES Estimates are from comparative interrupted time series regressions. Regressions for the likelihood of re-arrest are linear probability models. Regressions for the number of arrests are Poisson regression models. Each full sample regression is adjusted with gender and prior contact with the criminal justice system (in both the pre- and post-period) and an interaction between these variables and the running monthly counter to account for a time-varying relationship between the outcome and the covariates. The Midwest pair also adjusts for whether the arrest was a felony or misdemeanor and the interaction of this variable with the monthly counter. The Southwest county pair also adjusts for whether the arrest was for a parole violation and for whether the arrestee was Hispanic/Latino plus the interactions of these two variables with the monthly counter. Regressions using the Southeast county pair also adjust for whether the arrestee was African-American and the interaction of this variable with the monthly time trend.

A5. Comparison of individual-level and county-level CITS standard errors for estimates of the change in the probability of re-arrests and the number of arrests

	**Probability of Re-arrest**		**Number of Arrests**	
	Change in Intercept	*Change in Slope*	*Change in Intercept*	*Change in Slope*
**Midwest**				
Arrestee-level	0.11	0.007	0.038	0.002
County-level	0.57*	0.03*	0.006	0.0003
**Southwest**				
Arrestee-level	0.09	0.005	0.03	0.001
County-level	0.69	0.03	0.02	0.001
**Southeast**				
Arrestee-level	0.13	0.008	0.51	0.003
County-level	0.44	0.02	0.01	0.001

Sources/Notes: SOURCES Authors' analyses of arrest data from county jails. NOTES Estimates are from comparative interrupted time series regressions. Regressions for the likelihood of re-arrest are linear probability models. Regressions for the number of arrests are ordinary least squares regression models. The Midwest pair adjusts for whether the arrest was a felony or misdemeanor and the interaction of this variable with the monthly counter. The Southwest county pair also adjusts for whether the arrest was for a parole violation and for whether the arrestee was Hispanic/Latino plus the interactions of these two variables with the monthly counter. Regressions using the Southeast county pair also adjust for whether the arrestee was African-American and the interaction of this variable with the monthly time trend. *denotes that p-value becomes non-significant at the county-level analysis compared to the arrestee-level analysis.
